# Anti-Ageing Protein β-Klotho Rejuvenates Diabetic Stem Cells for Improved Gene-Activated Scaffold Based Wound Healing

**DOI:** 10.3390/jpm11010004

**Published:** 2020-12-22

**Authors:** Meenakshi Suku, Ashang Luwang Laiva, Fergal J. O’Brien, Michael B. Keogh

**Affiliations:** 1 Royal College of Surgeons in Ireland, Medical University of Bahrain, Kingdom of Bahrain P.O. Box 15503, Ireland; sukum@tcd.ie (M.S.); lluwang@rcsi-mub.com (A.L.L.); 2 Tissue Engineering Research Group, Department of Anatomy and Regenerative Medicine, Royal College of Surgeons in Ireland, 123 St. Stephen’s Green, Dublin 2, Ireland; fjobrien@rcsi.ie; 3 Trinity Centre for Bioengineering, Trinity Biomedical Sciences Institute, Trinity College Dublin, Dublin 2, Ireland

**Keywords:** gene-activated scaffolds, β-klotho, stem cells rejuvenation, adipose-derived stem cells, wound healing

## Abstract

Skin wounds can lead to serious morbidity complications in diabetic patients due to the reduced healing potential of autologous stem cells. One reason for the low functional potency of stem cells from diabetic patients (diabetic stem cells) is attributed to their senescent-like nature. Here, we investigated if an anti-ageing protein, β-klotho, could be used to rejuvenate diabetic stem cells and to promote pro-angiogenic gene-activated scaffold (GAS)-induced functional response for wound healing applications. Human stem cells derived from the adipose tissue (adipose-derived stem cells (ADSCs)) of normal and diabetic (type 2) donors were used for the study. We report that the β-klotho priming facilitated inflammatory signal pruning by reducing interleukin-8 release by more than half while concurrently doubling the release of monocyte chemoattractant protein-1. Additionally, β-klotho priming enhanced the pro-angiogenic response of diabetic ADSCs on GAS by dampening the release of anti-angiogenic factors (i.e., pigment epithelium-derived factor, tissue inhibitor of metalloproteinase-1 and thrombospondin-1) while simultaneously supporting the expression of pro-angiogenic factors (i.e., Vascular Endothelial Growth Factor (VEGF), angiopoietin-2 and angiogenin). Finally, we show that β-klotho pre-treatment expedites the cellular expression of matrix proteins such as collagen IV and collagen VI, which are implicated in tissue maturation. Taken together, our study provides evidence that the synergistic effect of the pro-angiogenic GAS and β-klotho activation effectively accelerates the functional development of diabetic ADSCs for wound healing applications.

## 1. Introduction

With the average life expectancy increasing worldwide, the prevalence of chronic, age-related diseases is also increasing. Diabetes is one of the most common age-related disorders in the US and is of growing concern globally [1]. Wound healing, an intricate process, is further complicated in diabetes with cells exhibiting a non-wound healing phenotype [2,3]. Diabetic wounds often show delayed wound healing response as a result of dysfunctional fibroblast and epidermal cells, failed angiogenesis and tissue maturation [4]. In hyperglycemic environments in vivo, stem cells have shown greater senescence, and adipose-derived stem cells (ADSCs) in particular have shown poor angiogenic properties, with reduced expression of stromal derived factor-1α (SDF-1α), Vascular Endothelial Growth Factor (VEGF), etc. [5].

The field of tissue engineering has brought forward a number of exciting possibilities to treat chronic wounds, including the first Food and Drug Administration (FDA)-approved collagen composite scaffold, developed by Integra^®^ Lifesciences (Integra Dermal Regeneration Template) [6,7]. However, there is a need for further research to obtain gold standard bioactive scaffolds for the treatment of chronic wounds. The application of therapeutics such as growth factor and gene delivery are prospective ways to achieve this. One potential avenue for treating chronic wounds is by entrapping therapeutic genetic material within a scaffold to obtain a gene-activated scaffold (GAS). The use of a GAS facilitates improved local production of the gene of interest by enabling cells to take up the gene from the scaffold, which thus acts as both a reservoir for the therapy as well as a template for tissue repair [8]. Furthermore, delivery of cell-seeded scaffolds into wound sites has been shown to allow for faster tissue regeneration [9]. Therefore, a cell-seeded GAS that particularly creates a biomimetic cell niche could be of great potential in the treatment of chronic wounds. One such GAS has recently been developed within our laboratory. It combines SDF-1α plasmid on a lyophilized collagen-chondroitin sulphate scaffold, which has high porosity and a proven potential in improving angiogenesis [10]. SDF-1α is a chemokine well known for its significant contribution in accelerating the process of wound healing and is severely depleted in diabetic wounds [11].

In addition to poor angiogenic properties, diabetic stem cells display impaired wound healing [12], increased cellular senescence and apoptosis [13]. Senescence of mesenchymal stem cells usually occur with in vitro cell culture [14], while in vivo, senescent cells accumulate in tissues with growing age and disrupt the structure and functions of the tissue because of the components they secrete, such as extracellular matrix (ECM)-degrading enzymes, inflammatory cytokines and growth factors [15]. Hyperglycaemia in diabetes can further aid to stem cell senescence [16]. Almost a decade ago, the clearance of senescent cells from a mouse model was found to delay the onset of age-related diseases [17], indicating the potential for development of novel treatments of these diseases. A common mode of activation of cellular senescence is the Wnt pathway, which has been proven to be triggered by the deficiency of a protein called klotho [18]. On deletion of this gene, phenotypes similar to ageing were observed in mice [19] and, conversely, the overexpression of klotho was shown to increase their lifespan [20]. The gene was found to encode a 130-kDa transmembrane protein that shares sequence homology with β-glucosidase and to bind to multiple fibroblast growth factor receptors (FGFRs) [21]. The klotho family is comprised of α-klotho, β-klotho and γ-klotho. β-klotho is expressed the most in adipose tissue and the pancreas [22]; it is also known to attenuate the IGF-1/insulin pathway [23], indicating relevance in the treatment of diabetes wounds. Therefore, it is thought that priming of diabetic stem cells with β-klotho could potentially invigorate them and improve the wound healing response when on SDF-GAS.

Adipose tissue represents a rich source for harvesting autologous stem cells. The stem cells in adipose tissue can be harvested using a minimally invasive liposuction approach. Moreover, the yield capacity of stem cells from adipose tissue (ADSCs) could be as high as 500 times that of stem cells derived from the same mass of bone marrow [24]. We, therefore, sought to utilize ADSCs as cell candidates to investigate the rejuvenating effects of the β-klotho protein and its synergism with SDF-1α gene-activated scaffold (SDF-GAS) in the functional activation of ADSCs. To that end, this project aims to revitalize diabetic ADSCs using β-klotho and to compare its early wound healing ability with treated and untreated normal and diabetic ADSCs on SDF-GAS.

## 2. Materials and Methods

### 2.1. SDF Plasmid Formulation and Preparation of Polyplex Vector

Previously reported techniques were followed for plasmid formulation and polyplex preparation [10]. In short, the plasmid DNAs (pDNA) encoding for SDF-1α (pSDF) were first amplified by transforming chemically competent DH5α *Escherichia coli* cells (Biosciences, Dublin, Ireland), in accordance with the manufacturer’s protocol. Transformed cells were selectively expanded over Lysogeny broth (LB) plates that contained 100 μg/mL of blasticidin as a selective antibiotic for pSDF. After 24 h at 37 °C, bacterial colonies were harvested and further amplified in LB broth containing blasticidin and cultured overnight in a shaker incubator at 37 °C. The plasmid purification was then carried out using a QIAGEN^®^ EndoFree^®^Plasmid Maxi kit (Qiagen, Manchester, UK), and the final nucleic acid concentration was determined using NanoDrop 1000 spectroscopy. Plasmids were further diluted in tris-ethylenediamine tetraacetic acid (TE) buffer to obtain a working concentration of 0.5 μg/μL and stored at −20 °C until use. Based on previous studies, polyplex particles were formulated by mixing a specified amount of branched cationic 25 kDa polyethyleneimine (PEI) (Sigma-Aldrich, Dublin, Ireland) and anionic pDNA (fixed at a dose of 2 μg) to give an N/P ratio of 10.

### 2.2. Expansion and Pre-Treatment of Normal and Diabetic ADSCs with β-Klotho

Lipoaspirate-derived ADSCs from a normal (female, age 33, Cat No. 10HU-001, Lot No. 200359) and a diabetic (type 2) patient (female, age 45, Cat no.10HU-007, Lot No. 200404) were purchased from iXCells Biotechnologies, San Diego, CA, USA. The ADSCs were received at passage 2 and expanded to passage 4 in a 1:1 Dulbecco’s Modified Eagles Medium/Nutrient (DMEM) mixture F-12 (D8437, Sigma-Aldrich, Gillingham, UK supplemented with 10% FBS (Gibco, Paisley, UK), 2% penicillin/streptomycin (Sigma-Aldrich, UK) and 1% amphotericin B (Gibco, UK). Normal ADSCs at passage 4 were seeded at a density of 8 × 10^3^ cells per well in 24-well adherent plates (Corning, Costar, UK) and treated with a range of concentrations of β-klotho from 10 µg/mL to 10 ng/mL (10 µg/mL, 5 µg/mL, 2 µg/mL, 1 µg/mL, 0.5 µg/mL, 0.25 µg/mL, 0.1 µg/mL, 0.05 µg/mL and 0.01 µg/mL) for 24 h to deduce the optimum concentration of β-klotho required to enhance the viability of the cells. The same study was repeated for diabetic ADSCs with a range of concentrations of β-klotho from 2 µg/mL to 10 ng/mL. The study was conducted over a period of 24 h, and untreated normal and diabetic ADSCs were used as the respective controls. Cell viability was assessed using the colorimetric [3-(4,5-dimethylthiazol-2-yl)-5-(3-carboxymethoxyphenyl)-2-(4-sulfophenyl)-2H-tetrazolium (MTS )assay (CellTiter 96^®^ Aqueous One Solution, Promega, Dane County, WI, USA). Briefly, after 24 h of the β-klotho pre-treatment, 20 µL of MTS reagent was added to 100 µL of media and incubated at 37 °C for 3 h. The intensity of the colour was measured using a plate reader (Multiskan Go Plate reader, Thermo Scientific, Basingstoke, UK) at an absorbance of 490 nm. The cells were counted using a cell counter (Countess II, Life Technologies, Paisley, UK). Images of the cells were captured using a phase contrast microscope (Olympus, Tokyo, Japan), and the cell number was counted after 24 h.

### 2.3. Development of SDF Gene-Activated Collagen-CS Scaffolds

The collagen-chondroitin sulphate scaffolds were prepared by using a previously developed freeze-drying method [25]. The scaffolds were dehydrothermally (DHT) treated at 105 °C for 24 h using a vacuum oven for both sterilization and crosslinking. The sterilized scaffolds with a thickness of approximately 4 mm were then cut to 8 mm diameter using a biopsy punch, followed by crosslinking with a mixture of 14 mM N-(3-Dimethylaminopropyl)-N′-ethylcarbodiimide hydrochloride (EDAC: Sigma-Aldrich, UK) and 5.5 mM N-Hydroxysuccinimide (NHS: Sigma-Aldrich, UK) to further structurally reinforce the scaffold. PEI-pDNA polyplexes were formulated as described in Section 2.1 and added to the scaffolds. The study was conducted as a comparison between normal and diabetic ADSCs treated with β-klotho (β-klotho^+^) and without β-klotho (β-klotho^−^) for 7 days. A total of 5 × 10^5^ cells (2.5 × 10^5^ per side), pre-treated for 24 h with optimal concentrations of β-klotho, were seeded onto the scaffold followed by the addition of 2 mL of OptiMEM (Gibco, UK), and the plates were incubated at 37 °C for 24 h, after which the media was removed and replaced with 2 mL of growth media. Scaffolds were cultured per group in triplicates, and β-klotho^−^ groups were used as the controls. Media were changed every 3 days, and conditioned media (CM) were stored at −20 °C for analysis.

### 2.4. Analysis of Soluble Factors

To determine the soluble angiogenic factors released on Day 7, CM collected from three replicates was pooled together and was analysed using a Human Proteome Profiling Kit (R & D Systems, Minneapolis, MN, USA) using methods adopted from previous studies [26,27,28]. Analysis of the soluble factors was performed in accordance with the manufacturer’s protocol. Briefly, the proteome profiler membranes were incubated with the blocking buffer on a rocking platform shaker for 1 h. The samples were prepared by adding 500 µL of CM to the array buffer, and 15 µL of the reconstituted detection antibody was added to the prepared sample. The sample was then incubated for 1 h at room temperature. The membranes were incubated with the sample/antibody mixture overnight at 2–8 °C on a rocking platform shaker. The following day, the membranes were removed and washed for 10 min each on the rocking platform shaker with 1× wash buffer three times. Streptavidin-horseradish peroxidase (HRP) diluted in array buffer 5 was added to the membranes and incubated in the rocking platform shaker for 30 min, followed by three 10 min washes with 1× wash buffer. The membranes were finally exposed to the chemireagent mix for 1 min before viewing using the Chemidoc XRS system to detect the light produced at each spot in proportion to the analyte bound. The soluble factors of interest are listed in Table 1.

### 2.5. Quantitative Real-Time Polymerase Reaction (qRT-PCR) Analysis of Normal and Diabetic ADSCs on SDF-GAS

In order to study the early functional activation of normal and diabetic ADSCs post 24 h β-klotho pre-treatment, the cells from the scaffolds were harvested on day 7 for analysis. The RNA was extracted after lysing the cells on the scaffold using the Qiazol reagent (Qiagen, UK). Chloroform was then added to the cell lysate to separate it into the protein, DNA and RNA phases. RNA was extracted using the RNeasy Kit (Qiagen, UK). Prior to using a reverse transcriptase enzyme (Qiagen, UK) for cDNA synthesis, genomic DNA was removed by heating the RNA to 42 °C for 2 min using a genomic DNA wipe-out buffer (Qiagen, UK). qRT-PCR was then performed on cDNA using the primer (Qiagen, UK) CXCL12 (SDF-1α). Fold change in the mRNA expression in relation to the untreated control on day 7 was calculated using the 2^−∆∆CT^ method [38] from averages of 3 samples for each group. Glyceraldehyde 3-phosphate dehydrogenase (GAPDH) (Hs_GAPDH_1_SG) was used as the housekeeping gene.

### 2.6. Immunofluorescent Imaging of Normal and Diabetic ADSCs on SDF-GAS and Quantification of Expressed Proteins

The scaffolds harvested on day 7 were first washed with PBS and fixed in 10% neutral buffered formalin for 10 min and then processed using the standard protocol for paraffinization. Eight-μm thick slices were obtained, which were deparaffinized and mounted on charged slides. The cells were permeabilized prior to staining, with 0.2% Tween^®^20 (Sigma-Aldrich, Saint-Quentin Fallavier, France) solution in 1× PBS for 30 min and blocked using 10% NGS (Normal Goat Serum, Invitrogen, Paisley, UK)/5% Bovine serum albumin (BSA)/0.3 M glycine (prepared in permeabilizing solution) for 1 h to inhibit nonspecific protein interaction. Subsequently, primary antibodies diluted in 1% BSA in PBS were added and incubated at 4 °C overnight. The primary antibodies (Abcam, Cambridge, UK) used for staining were SDF-1α (1:100), CXCR7 (1:50), fibronectin (1:100), collagen IV (1:100) and collagen VI (1:100).

The following day, the slides were washed in PBS thrice for 2 to 3 min each and incubated with either Alexa 488-conjugated goat anti-mouse IgG (Invitrogen, UK) or Alexa 594-conjugated goat anti-rabbit IgG (A11012, Invitrogen, UK), in accordance with the primary antibody at 1:800 dilution at room temperature for 1 h in dark. The slides were then rinsed again using PBS, and the nuclei was stained using the fluoroshield mounting medium with 4′,6-diamidino-2-phenylindole (DAPI) (Abcam, UK) and covered with coverslips. The slides were then imaged using a fluorescence microscope (Olympus BX43, Japan). All images were captured at 40× magnification. The images were semi-quantified using ImageJ.

### 2.7. Statistical Analysis

All results are expressed as mean ± standard deviation. One-way and two-way ANOVAs as well as *t*-Tests were used to demonstrate the statistical significance between groups, where *p* < 0.05 was considered significant.

## 3. Results

### 3.1. β-Klotho Pre-Treatment Increased the Proliferation and Viability of Normal and Diabetic ADSCs in 2D

**Figure 1:**
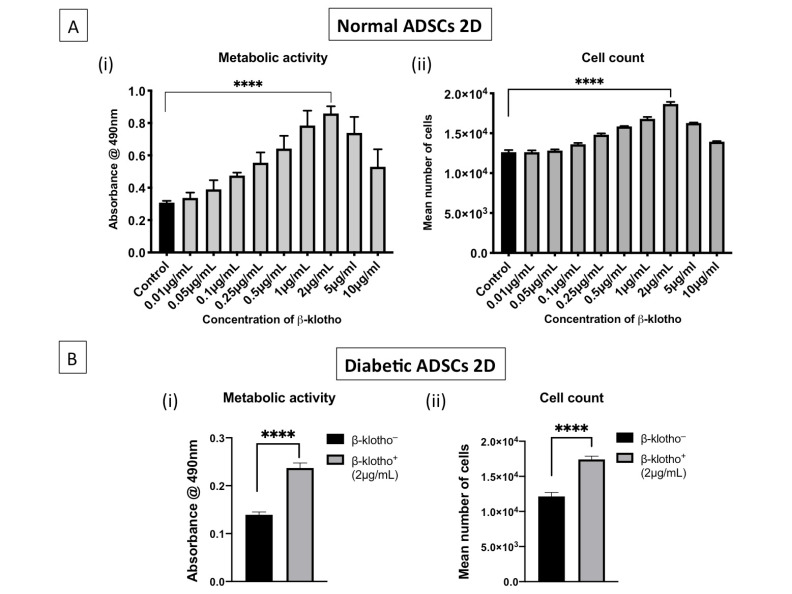
Metabolic activity and cell count of normal and diabetic adipose-derived stem cells (ADSCs) after 24 h of β-klotho treatment in 2D: (**A**) the effect of β-klotho treatment in normal ADSCs. β-klotho at a concentration of 2 µg/mL was found to be optimal for enhancing the growth of normal ADSCs. Treatment with 2 µg/mL β-klotho significantly enhanced the metabolic activity (*p* < 0.0001) as well proliferation (*p* < 0.0001) of normal ADSCs compared to the non-treated control. (**B**) Diabetic ADSCs treated with 2 µg/mL β-klotho also displayed a significantly higher metabolic activity (i) as well as proliferation (ii) than the non-treated control (*p* < 0.0001). **** indicates statistical significance of *p* < 0.0001. β-klotho^+^ and β-klotho^−^ refers to β-klotho primed ADSCs and untreated controls respectively.

The MTS assay was first performed on normal ADSCs to assess their viability after exposure to various concentration of β-klotho (0.01 µg/mL to 10 µg/mL) in 2D. Two µg/mL of β-klotho were found to significantly improve the viability of the cells with *p* < 0.0001 (Figure 1A). Analogous to cell viability, cell count showed over two-fold increase within 24 h in 2 µg/mL β-klotho-treated normal ADSCs (Figure 1A(ii)). Observing the response of normal ADSCs to 2 µg/mL of β-klotho, this concentration was primarily analysed in diabetic ADSCs. The MTS assay performed on diabetic ADSCs pre-treated with 2 µg/mL β-klotho for 24 h in cell culture plates showed a significantly higher viability in comparison to the untreated control (Figure 1B(i)). The same trend was observed in cell count, with 2 µg/mL β-klotho-treated diabetic ADSCs showing a two-fold increase in cell proliferation in 24 h (Figure 1B(ii)).

### 3.2. β-Klotho Did Not Impede the Expression of SDF-1α and CXCR7

**Figure 2:**
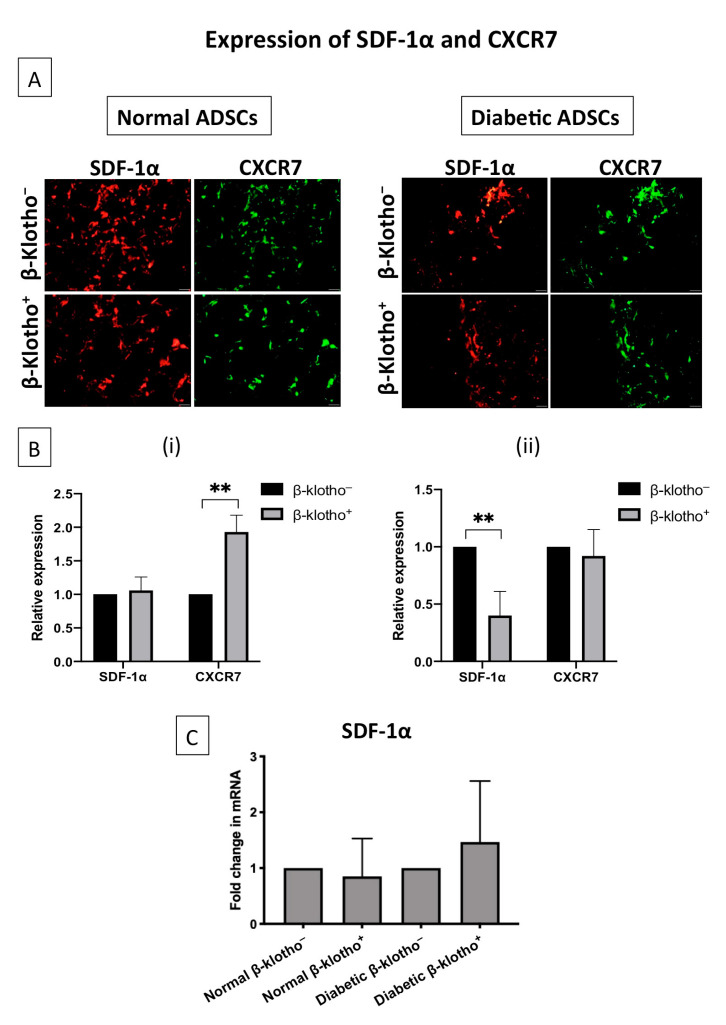
Expression of SDF-1α and CXCR7 in normal and diabetic ADSCs cultured on stromal derived factor-1α gene-activated scaffold (SDF-GAS): (**A**) representative immunofluorescence images showing the expression of SDF-1α and CXCR7 in β-klotho^+^ normal ADSCs in comparison to control groups; (**B**) semi-quantified relative expression of SDF-1α and CXCR7 in β-klotho^+^ normal ADSCs and diabetic ADSCs, where (i) β-klotho^+^ ADSCs showed a higher CXCR7 expression than its control (mean ± SD, *p* < 0.01) and (ii) β-klotho^+^ diabetic ADSCs showed a lower SDF-1α expression than its control (mean ± SD, *p* < 0.01); and (**C**) gene expression analysis of SDF-1α gene expression between β-klotho-treated and untreated ADSCs. Scale bar, 20 µm. ** indicates statistical significance of *p* < 0.01. β-klotho^+^ and β-klotho^−^ refers to β-klotho primed ADSCs and untreated controls respectively.

The effect of β-klotho on SDF-1α transfection was assessed by verifying the expression of SDF-1α and its downstream regulator CXCR7. A preliminary gene expression analysis showed that there was no significant difference in the expression of the SDF-1α gene between the β-klotho^+^ and β-klotho^−^ ADSCs (Figure 2C). However, protein expression analysis showed that β-klotho treatment did not affect the expression of SDF-1α protein in normal ADSCs (Figure 2B(i)) but downregulated the expression of SDF-1α protein by 2-fold in diabetic ADSCs (Figure 2B(ii)). With regards to CXCR7 expression, β-klotho priming upregulated the expression of CXCR7 by almost 2-fold in normal ADSCs while the expression remained unaffected in diabetic ADSCs (Figure 2B).

### 3.3. β-Klotho Pre-Treatment Reduced the Production of Fibrinogenic Factor Plasminogen Activator Inhibitor-1 and Attenuated Interleukin-8 Production in Diabetic ADSCs on SDF-GAS

**Figure 3:**
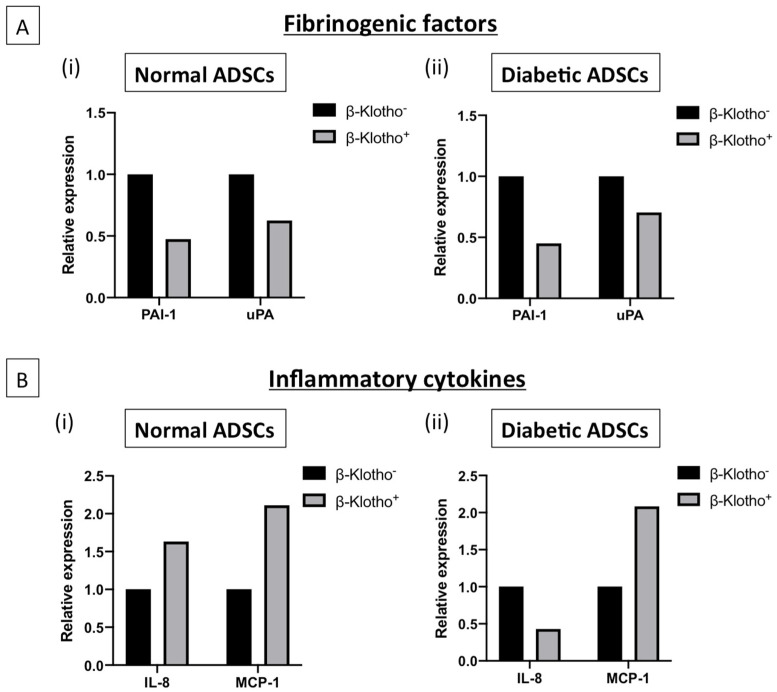
The expression of soluble fibrinogenic and inflammatory factors in normal and diabetic ADSCs cultured on SDF-GAS is shown. (**A**) Relative expression of fibrinogenic factors: Overall, β-klotho^+^ ADSCs showed downregulation of fibrinogenic factors compared to its non-treated controls. β-klotho priming markedly reduced (approximately 2-fold) the production of plasminogen activator inhibitor-1 in both (i) normal and (ii) diabetic ADSCs. (**B**) Relative expression of inflammatory cytokines: (i) β-klotho priming enhanced the production of both interleukin-8 and monocyte chemoattractant protein-1 in normal ADSCs. (ii) β-klotho primed diabetic ADSCs showed downregulation of interleukin-8 while upregulating the production of monocyte chemoattractant protein-1 by ~2-fold compared to the control group. β-klotho^+^ and β-klotho^−^ refers to β-klotho primed ADSCs and untreated controls respectively.

β-klotho^+^ cells were found to downregulate the production of plasminogen activator inhibitor-1 by two-fold, marking increased potential for fibrin breakdown than in the β-klotho^−^ controls (Figure 3A). Although normal ADSCs displayed an increased expression of interleukin-8 with β-klotho priming, monocyte chemoattractant protein-1 release was upregulated compared to its control (Figure 3B(i)). Conversely, β-klotho pre-treatment reduced interleukin-8 release in diabetic ADSCs by 55% while simultaneously doubling the monocyte chemoattractant protein-1 release (Figure 3B(ii)).

### 3.4. β-Klotho Pre-Treatment Facilitated Sustained Expression of Angiogenic Factors and Decreased the Expression of Anti-Angiogenic Factors in Diabetic ADSCs on SDF-GAS

**Figure 4:**
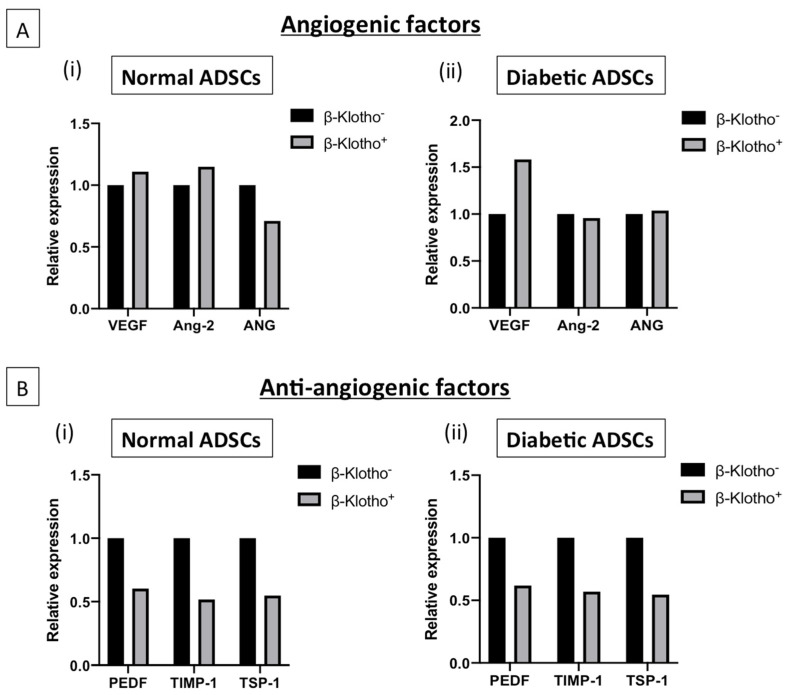
The relative expression of soluble angiogenic and anti-angiogenic factors in normal and diabetic ADSCs cultured on SDF-GAS is shown. (**A**) Relative expression of the angiogenic factors: (i) β-klotho priming mildly upregulated the production of VEGF and angiopoietin-2 but downregulated the production of angiogenin by 29% in normal ADSCs compared to the untreated control. Meanwhile, (ii) β-klotho primed ADSCs showed a marked increase (56%) in the production of pro-angiogenic factor VEGF than the untreated diabetic ADSCs. (**B**) Relative expressions of the anti-angiogenic factors- pigment epithelium-derived factor, tissue inhibitor of metalloproteinase-1 and thrombospondin-1. Figures (i) and (ii) show that β-klotho primed ADSCs exhibit considerably reduced production of the anti-angiogenic factors compared to their respective controls. β-klotho^+^ and β-klotho^−^ refers to β-klotho primed ADSCs and untreated controls respectively.

β-klotho priming did not impact the pro-angiogenic profile of normal ADSCs on SDF-GAS considerably (Figure 4A(i)). However, strong downregulation was found in the expression of anti-angiogenic factors (Figure 4B(i)). VEGF release in β-klotho^+^ normal ADSCs was increased by 9%. However, angiopoietin-2 increased by 13% and angiogenin decreased by 29%, potentially indicating the conclusion of angiogenesis. Nonetheless, anti-angiogenic factors such as pigment epithelium-derived factor, tissue inhibitor of metalloproteinase-1 and thrombospondin-1 were downregulated. Figure 4A(ii) shows that β-klotho^+^ diabetic ADSCs exhibited sustained expression of angiogenic factors in comparison to the control group, with 56% upregulation in VEGF and a fairly consistent expression of angiopoietin-2 and angiogenin. In a normal physiological environment, angiogenin is expected to be significantly lower than VEGF, and this was found to be positive in both normal and diabetic ADSCs. Additionally, β-klotho^+^ diabetic ADSCs showed a decrease in the expression of anti-angiogenic factors pigment epithelium-derived factor, tissue inhibitor of metalloproteinase-1 and thrombospondin-1 (Figure 4B(ii)). Overall, sustained angiogenic response was noted in β-klotho primed cells.

### 3.5. β-Klotho Pre-Treatment Improved the Expression of ECM Proteins in Diabetic ADSCs on SDF-GAS

**Figure 5:**
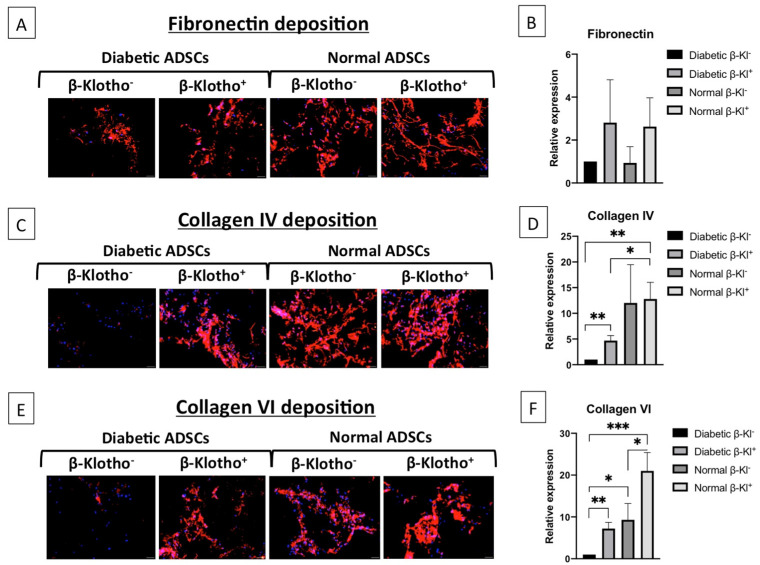
Analysis of fibronectin, collagen IV and collagen VI deposition in normal and diabetic ADSCs cultured on SDF-GAS: (**A**,**C**,**E**) representative images of fibronectin, collagen IV and collagen VI matrix depositions by the ADSCs at day 7, respectively. Nuclei are stained in blue. (**B**,**D**,**F**) The relative expressions of the matrix proteins fibronectin, collagen IV and collagen VI, respectively: (**B**) Semi-quantitative analysis of fibronectin did not show any significant differences between the groups. (**D**) Relative to the untreated diabetic ADSCs; both β-klotho^+^ normal and diabetic ADSCs demonstrated significantly higher deposition of basement membrane protein collagen IV at day 7. (**F**) β-klotho^+^ ADSCs also deposited significantly higher amounts of collagen VI than the untreated diabetic ADSCs. Scale bar, 20 µm. *, ** and *** indicate statistical significance of *p* < 0.05, *p* < 0.01 and *p* < 0.005 respectively.

After examining the secreted factors, we sought to analyse the ECM protein deposition in normal and diabetic ADSCs after β-klotho priming, cultured on SDF-GAS. Fibronectin forms a provisional matrix during the initial stages of wound healing to facilitate matrix deposition [39], and its expression was comparable in normal ADSCs irrespective of β-klotho pre-treatment (Figure 5A,B). Collagen IV, which is an essential basement membrane protein, was comparable in β-klotho^+^ normal ADSCs and controls (Figure 5C,D). Nonetheless, collagen VI expression was upregulated by over two-fold in β-klotho^+^ normal ADSCs (Figure 5E,F). In diabetic ADSCs, although β-klotho priming did not affect the fibronectin deposition (Figure 5A,B), collagen IV and collagen VI depositions were increased by over four-fold (Figure 5C,D) and six-fold (Figure 5E,F), respectively. Collagen VI expression was generally found to be greater in normal ADSCs than in diabetic ADSCs (Figure 5F). However, β-klotho^+^ diabetic ADSCs and β-klotho^−^ normal ADSCs did not have any significant difference in the expression of fibronectin, collagen IV and collagen VI.

## 4. Discussion

In this study, we explored the functional growth of β-klotho-rejuvenated diabetic ADSCs on a pro-angiogenic stromal-derived factor-1α gene-activated scaffold as an approach to generate an autologous stem cell-based therapeutic strategy. β-klotho priming was found to attenuate the inflammatory response in diabetic ADSCs by reducing interleukin-8 release by more than half without compromising the monocyte chemoattractant protein-1 release. The pro-angiogenic response was observed to be enhanced in β-klotho^+^ cells on SDF-GAS with a downregulation of anti-angiogenic proteins (pigment epithelium-derived factor, tissue inhibitor of metalloproteinase-1 and thrombospondin-1) together with simultaneous sustained expression of pro-angiogenic proteins (VEGF, angiopoietin-2 and angiogenin). Finally, early cellular expressions of collagen IV and collagen VI were achieved by β-klotho priming, indicating an expedited wound healing response in cells. Taken together, these results indicate that the synergistic effect of the pro-angiogenic GAS and β-klotho activation effectively accelerates the functional growth of diabetic ADSCs for wound healing applications.

Wound healing is an efficiently orchestrated event that has a number of distinct yet overlapping phases that carry out complex processes (haemostasis, inflammation, proliferation and maturation) to return the affected tissue to homeostasis [40]. Stem cells mobilize from the bone marrow and coordinate healing processes through the production of multiple signalling factors [41,42]. However, cellular senescence can extensively alter the production of signalling factors that can lead to impaired healing response [43]. Diabetic stem cells are primarily known to be senescent [5], which is further increased by hyperglycaemia, ultimately causing stem cells apoptosis [16]. Cellular senescence is a challenge that has yet to be overcome to improve graft survival and to enhance healing with tissue engineering approaches [44].

Klotho is one of the therapeutic factors that can rescue cells from undergoing senescence and that can improve their functions [20]. Relative to the α-isoform, little is known about the effect of β-klotho on stem cells and particularly diabetic ADSCs. Nevertheless, studies have shown that obesity causes reduced expression of β-klotho in the adipose tissue [45] and that deficiency of β-klotho impairs ADSC functions through alterations in telomerase activity [46]. Therefore, we hypothesized that priming diabetic ADSCs with β-klotho may improve the therapeutic effect of diabetic ADSCs. As anticipated, we noted that β-klotho significantly enhanced the metabolic activity as well as proliferation of diabetic ADSCs. In 24 h, β-klotho-primed ADSCs showed a significant 45% increase in cell proliferation compared to untreated controls. This β-klotho-induced rapid cellular proliferation may be beneficial in the shortening cell culture time for generation of large tissue grafts for autologous transplantation.

Cell-free scaffolds such as the collagen-chondroitin sulphate (coll-CS) scaffold have proven its efficacy in facilitating skin regeneration since 1980s [47] and are now available in the market. This scaffold facilitates cell infiltration and has shown positive results in clinical trials for burns [48], scars [49] and more recently, diabetic foot ulcers (DFUs) [7]. This scaffold can further be functionalized with pro-angiogenic genes to enhance their bioactivity. Recently, we showed that the coll-CS scaffold functionalized with a pro-angiogenic SDF-1α gene could enhance pro-angiogenic responses in bone marrow stem cells as well as neuronal human Schwann cells [10,50]. However, it remains to be seen if diabetic stem cells can use this GAS template to enhance their functional properties.

The inflammatory phase of wound healing is usually marked by cellular expression and release of a number of pro-inflammatory cytokines. Although the presence of inflammatory cytokines is essential in normal wound healing, persistent and prolonged expression of these cytokines could be detrimental [51]. The presence of high amounts of interleukin-8 is a hallmark in the ADSCs of patients suffering from diabetes, with the expression of interleukin-8 being almost two-fold greater than that of monocyte chemoattractant protein-1 [52]. To drive the healing process, the production of monocyte chemoattractant protein-1 is essential [53]. In this study, we show that β-klotho priming facilitated enhanced production of monocyte chemoattractant protein-1 in diabetic ADSCs, suggesting improved regulation of inflammatory signalling in diabetic ADSCs.

During the inflammatory phase, a number of angiogenic factors are also released to promote neo-vascularization [54]. One of the most important angiogenic stimulants is the VEGF, which is also a downstream regulator of SDF-1α [55]. Our study shows that VEGF is strongly induced only in the diabetic ADSCs with an increase over 56% compared to its untreated equivalent. Additionally, β-klotho priming negligibly affected the production of angiopoietin-2, which is known to work in concert with VEGF to regulate microvascular permeability and integrity [56]. This angiogenic state characterized by increased VEGF production paired with reduced angiopoietin-2 is considered crucial for proper induction of angiogenesis [57].

Matrix deposition is another key feature essential for efficient integration of the graft with host tissue [58]. Therefore, we examined the effect of β-klotho on the deposition of primary matrix proteins of the basement membrane by ADSCs. Fibronectin is one of the first matrix proteins deposited by cells and serves as a provisional matrix for subsequent collagen deposition [59]. Collagen IV represents a major component of the basement membrane and is essential for stable maturation of cells [60], while collagen VI anchors other matrix proteins such as fibronectin and collagen IV to regulate cell motility [61]. These ECM components can directly influence cell growth, differentiation and tissue regeneration [62]. Moreover, an increase in collagen deposition is a feature of enhanced healing in vivo [63]. Accordingly, given our finding that β-klotho priming significantly enhances matrix deposition by ADSCs, it is plausible that the application of β-klotho-primed ADSCs with SDF-GAS will lead to accelerated healing in vivo.

Future iteration of this work will focus on understanding the pathways activated by β-klotho to exert rejuvenating effects on diabetic ADSCs and application of these cells in vivo. Nevertheless, these encouraging results on β-klotho-primed ADSCs on SDF-GAS can be expected to boost further studies to bring forward clinically translatable applications.

## 5. Conclusions

The functional impairment and senescence of diabetic ADSCs is detrimental to wound healing, and here, we show that it is feasible through β-klotho priming to rejuvenate diabetic ADSCs to improve their functional ability using an SDF-1α gene-activated collagen-chondroitin sulphate scaffold. The synergistic effect of β-klotho pre-treatment and SDF-GAS could be of great therapeutic use in accelerating diabetic wound healing, facilitating normal wound repair conditions and increasing graft longevity. Therefore, in conclusion, this study shows that rejuvenation priming of diabetic stem cells with β-klotho on SDF-GAS could be an efficient bio-instructive system to generate new tissue by accelerating the process of wound healing in diabetic patients.

## Figures and Tables

**Table 1:** Soluble factors and its role in angiogenic wound healing.

Soluble Factor		Function
Fibrinogenic factors	Plasminogen Activator Inhibitor-1 (PAI-1)	Impedes fibrinolysis [29]
	Urokinase Plasminogen Activator (uPA)	Promotes fibrinolysis (antagonist to PAI-1) [30]
Inflammatory Cytokines	Interleukin-8 (IL-8)	Inflammatory response, neutrophil recruitment to the wound site [31]
	Monocyte Chemoattractant Protein-1 (MCP-1)	Inflammatory response, monocyte recruitment to the wound site [32]
Angiogenic factors	Vascular Endothelial Growth Factor (VEGF)	Early stimulation of angiogenesis [33]
	Angiogenin (ANG)	Stimulation of angiogenesis (late stage)
	Angiopoietin-2 (Ang-2)	Obstructs neo-vascularization, blood vessel maturation [34]
Anti-angiogenic factors	Pigment Epithelium Derived Factor (PEDF)	Promotes vessel regression [35]
	Tissue Inhibitor for Metalloproteinase-1 (TIMP-1)	Inhibits Matrix Metalloproteinases (MMPs) that break down the ECM to facilitate angiogenesis [36]
	Thrombospondin-1 (TSP-1)	Delays angiogenesis, poor vascularization [37]

## Data Availability

The data supporting this study are available on request from the authors.
